# Controlling Highly Pathogenic Avian Influenza, Bangladesh

**DOI:** 10.3201/eid1812.120635

**Published:** 2012-12

**Authors:** Shankar P. Mondal, David Tardif-Douglin, Robert Ryan-Silva, Rich Magnani

**Affiliations:** Development Alternatives Inc., Bethesda, Maryland, USA (S.P. Mondal, D. Tardif-Douglin, R. Ryan-Silva, R. Magnani);; and University of Minnesota, St. Paul, Minnesota, USA (S.P. Mondal)

**Keywords:** Highly pathogenic avian influenza, HPAI, subtype H5N1, influenza, poultry value chain, biosecurity, live bird markets, poultry farms, cleaning and disinfection, transmission, intervention, viruses, zoonotic diseases, Bangladesh

**To the Editor:** Highly pathogenic avian influenza (HPAI) A(H5N1) virus is a deadly zoonotic pathogen. Since 2003, HPAI infections have been reported in millions of poultry and wild birds from 63 countries (*1*) and in 598 humans, among whom there have been 352 reported deaths in 15 countries (*2*). HPAI (H5N1) virus is endemic in Bangladesh, and the first outbreak occurred in March 2007. Since then, the virus has spread to 49 of 64 districts in Bangladesh, and samples from 536 farms have tested positive for the virus. Bangladesh now ranks among countries worldwide with the highest reported number of HPAI outbreaks (*1*). Intermittent outbreaks in Bangladesh and clusters of disease across the border in northeastern India are dramatic reminders that the emergence of new, mutant viruses in developing countries could lead to a pandemic among humans. Six cases of nonfatal HPAI (H5N1) infection have been reported in Bangladesh (*2*). Live bird markets that are in poor physical condition and that lack or have poor biosecurity are probable sources of HPAI transmission to humans and for bird-to-bird transmission (*3*–*5*).

In 2008, a global project of the United States Agency for International Development, Stamping Out Pandemic and Avian Influenza (STOP AI), was initiated in Bangladesh. The project began with biosecurity training for veterinarians and livestock science graduates on some large-scale commercial farms. The local STOP AI office was established in Dhaka, the capital of Bangladesh, in February 2009, and the organization managed the project through its completion in September 2010 (Technical Appendix Figure 1). STOP AI initially organized 7 highly successful live bird market biosecurity training programs in 5 geographic divisions of Bangladesh; later, STOP AI piloted cleaning and disinfection activities in 2 live bird markets, Mohammadpur and Kaptan Bazaar, in Dhaka by working closely with the United Nations’ Food and Agriculture Organization. The Food and Agriculture Organization subsequently conducted cleaning and disinfection activities in 24 other markets within Dhaka and other districts in Bangladesh.

We focused on understanding the inter-relationships among household poultry producers, commercial farmers, suppliers, transporters, processors, and consumers that facilitate the process of producing and moving poultry, i.e., the entire poultry value chain (PVC). We describe how improved biosecurity on poultry farms and hygienic standards in live bird markets can reduce HPAI outbreaks. In resource-limited countries, like Bangladesh, these improvements can be made through training, technical support, financial assistance for infrastructure renovations, and incentive-driven trust-building between service providers and key PVC stakeholders. To determine whether interventions would reduce the number of HPAI infections, we implemented changes during 2009–2010 in 2 districts in Bangladesh, Gazipur and Dinajpur, that had a high number of cases (Technical Appendix Figure 2).

Using field-tested questionnaires, we conducted a baseline survey during in-person interviews with 1,372 poultry stakeholders (Table). Stakeholder workshops were held in each district to share survey findings and design biosecurity improvement programs. STOP AI implemented biosecurity training for 1,319 people in 53 subsector-specific 1-day sessions in Gazipur and Dinajpur (Technical Appendix Table). We created biosecurity improvement models (e.g., farm boundary, footbath) in 12 commercial farms in Gazipur and selected 2 live bird markets in each district for infrastructure improvements, including biogas and compost plants, that were needed for the cleaning and disinfection activities (Technical Appendix Figure 3). We provided technical support and <25% ($750) of the cost for each farm and <50% ($10,000) for each market on a cost-sharing basis.

**Table:** Analysis of pre- and postintervention survey data for biosecurity practices for HPAI (H5N1) virus in Gazipur and Dinajpur districts, Bangladesh, 2009–10*

Biosecurity practice	No. persons surveyed (% aware of practice; 95% CI)				
	Gazipur			Dinajpur	
	Baseline survey, n = 821	Final survey, n = 300†		Baseline survey, n = 525	Final survey, n = 209†
Awareness of bird deaths caused by HPAI	191 (23; 21–26)	219 (73; 68–78)		116 (22; 19–26)	88 (42; 36–49)
Awareness of bird culling resulting from HPAI	163 (20; 17–23)	186 (62; 56–67)		56 (11; 8–14)	47 (22; 17–29)
Awareness of HPAI cases among humans	138 (17; 14–20)	172 (57; 52–63)		21 (4; 3–6)	113 (54; 47–61)
Understand how HPAI is spread					
Do not know how HPAI is spread	209 (25; 23–29)	42 (14; 11–18)		286 (54; 50–59)	21 (10; 7–15)
Perceive that wild birds are the cause	466 (57; 53–60)	207 (69; 64–74)‡		134 (26; 22–29)	154 (74; 67–80)
Recognize sick poultry as a vector	43 (5; 4–7)	140 (47; 41–52)		53 (10; 8–13)	61 (29; 23–36)
Awareness of how to protect people					
Wear masks	190 (23; 20–26)	204 (68; 63–73)		44 (8; 6–11)	94 (45; 38–52)
Wear gloves	122 (15; 13–17)	136 (45; 40–50)		54 (10; 8–13)	75 (36; 30–43)
Wash hands	207(25; 23–29)	166(55; 50–60)		58 (11; 9–14)	139 (67; 60–73)
Kids should not handle birds	3 (0; 0.1–1)	203 (68; 62–73)		1 (0; 0.01–1)	54 (26; 20–32)
No need to protect	243 (30; 27–33)	0 (0)		294 (56; 52–60)	4 (2; 0.6–5)
Awareness of how to protect birds					
Separate chickens and ducks	28 (3; 3–4)	92 (31; 26–36)		18 (3; 2–5)	36 (17; 13–23)
Clean and disinfect poultry cages	288 (35; 32–38)	182 (61; 55–66)		39 (7; 5–10)	99 (47; 41–54)
Restrict entry to farms	226 (28; 25–31)	131(44; 38–49)		56 (11; 8–14)	56 (27; 21–33)
Vaccinate against Newcastle disease	12 (1; 0.8–2)	67 (22; 18–27)		7 (1; 0.6–3)	47 (22; 17–29)
Properly dispose of feces	158 (19; 17–22)	79 (26; 22–32)§		16 (3; 2–5)	117 (56; 49–63)
Wear proper clothing	38 (5; 3–6)	67 (22; 18–27)		29 (6; 4–9)	43 (21; 16–27)
Clean and disinfect transport vehicles	35 (4; 3–6)	105 (35; 30–40)		10 (2; 1–4)	20 (10; 6–14)
Keep dogs and cats away from farms	92 (11; 9–14)	99 (33; 28–39)		22 (4; 3–6)	33 (16; 11–21)
Do not know	170 (21; 18–24)	46 (15; 12–20)¶		267 (51; 47–55)	2 (1; 0.04–4)
Bird purchase preference					
Dead bird (slaughtered at market)	152 (19; 16–21)	136 (45; 40–51)		50 (10; 7–12)	70 (33; 27–40)
Live bird (slaughtered at home)	652 (79; 77–82)	167 (56; 50–61)		484 (92; 90–94)	136 (65; 58–71)

*HPAI, highly pathogenic avian influenza. †Two-sided χ^2^ test of significance compared with baseline data had p value of <0.0001, except as noted. ‡p = 0.0002. §p = 0.013. ¶p = 0.049.

After completion of all interventions, we conducted a final survey of 514 poultry stakeholders, including 70% of the original trainees from both districts (Table). We analyzed pre- and post-intervention survey data by using GraphPad Software (www.graphpad.com/quickcalcs/index.cfm). The results indicated that awareness of the proper disposal of birds that were culled or died because of HPAI had increased in both districts (p<0.0001); awareness of human HPAI cases rose substantially (p<0.0001); an understanding of how HPAI is spread (e.g., through sick or wild birds) changed (p<0.001); use of personal protective equipment (masks, gloves) and other precautionary measures (washing hands) increased (p<0.0001); awareness of protecting birds from HPAI (e.g., separately housing chickens and ducks) increased (p<0.05); and a preference for purchasing slaughtered birds instead of live birds at the markets increased (p<0.0001).

Substantially fewer HPAI outbreaks were reported and no clusters of infection were found during our intervention, 2009–2010 (Technical Appendix Figure 1), probably indicating that control measures were effective. The challenge now is to sustain the progress that has been made. Several months after completion of the STOP AI interventions, their effect on the incidence of disease in Bangladesh was limited. However, STOP AI could not be expected in the short term to dramatically reduce the high incidence of HPAI in Bangladesh. We have progressively and dramatically increased the scope and benefits of our pilot PVC implementation program, but additional work is needed. To help spread PVC approaches throughout the country, community leaders, imams of local mosques, and school teachers can be trained to implement awareness programs on safe practices for raising poultry and regular cleaning and disinfection of live bird markets. The strengthening of biosecurity measures will help control the spread of HPAI virus and other zoonotic diseases.

Technical Appendix Poultry subsector–specific biosecurity training in Gazipur and Dinajpur districts; key events of Stamping Out Pandemic and Avian Influenza project and weekly outbreaks of highly pathogenic avian influenza (HPAI) subtype H5N1 virus; intervention district locations and HPAI outbreaks in Gazipur and Dinajur; and infrastructure improvements to live bird markets in Dinajpur and Gazipur districts, Bangladesh.
