# Supplementary material for: Controlling Highly Pathogenic Avian Influenza, Bangladesh
Source: Emerg Infect Dis. 2012 Dec;18(12):2083–5. doi: 10.3201/eid1812.120635 (PMC3557891; doi:10.3201/eid1812.120635)
Supplement: Technical Appendix — Poultry subsector–specific biosecurity training in Gazipur and Dinajpur districts; key events of Stamping Out Pandemic and Avian Influenza project and weekly outbreaks of highly pathogenic avian influenza (HPAI) subtype H5N1 virus; intervention district locations and HPAI outbreaks in Gazipur and Dinajur; and infrastructure improvements to live bird markets in Dinajpur and Gazipur districts, Bangladesh. [file 12-0635-Techapp-s1.pdf]

Article DOI: http://dx.doi.org/10.3201/eid1812.120635

Controlling Highly Pathogenic Avian Influenza, Bangladesh

Technical Appendix

Table. Poultry subsector–specific biosecurity training in Gazipur and Dinajpur districts, Bangladesh, 2010*

	Gazipur		Dinajpur		Total	
Category	Sessions	People	Sessions	People	Sessions	People
Commercial farmers	8	200	6	150	14	350
Hatchery owners	1	21	1	25	2	46
Poultry transporters	5	125	6	150	11	275
Live bird market vendors	4	98	6	150	10	248
Backyard producers	4	100	12	300	16	400
**Total**	**22**	**544**	**31**	**775**	**53**	**1319**

*Each training session covered the following topics: an introduction to avian influenza; basic principles of biosecurity; biosecurity measures for specific subgroup; cleaning and disinfection principles; and biosecurity planning for specific subsector (replaced by “public health importance” for backyard subsector). All materials were translated into Bengali. Each of the training sessions had a capacity of 25 participants and each backyard session contained ≥40% woman participants. A demonstration for backyard producers included thorough cleaning and disinfection of a backyard poultry house done by the trainer assisted by a female participant. We learned that virtually all respondents who received our training tried to implement biosecurity practices they learned, but backyard farmers tended to adopt more biosecurity practices than other beneficiaries, and women tend to adopt more than men.

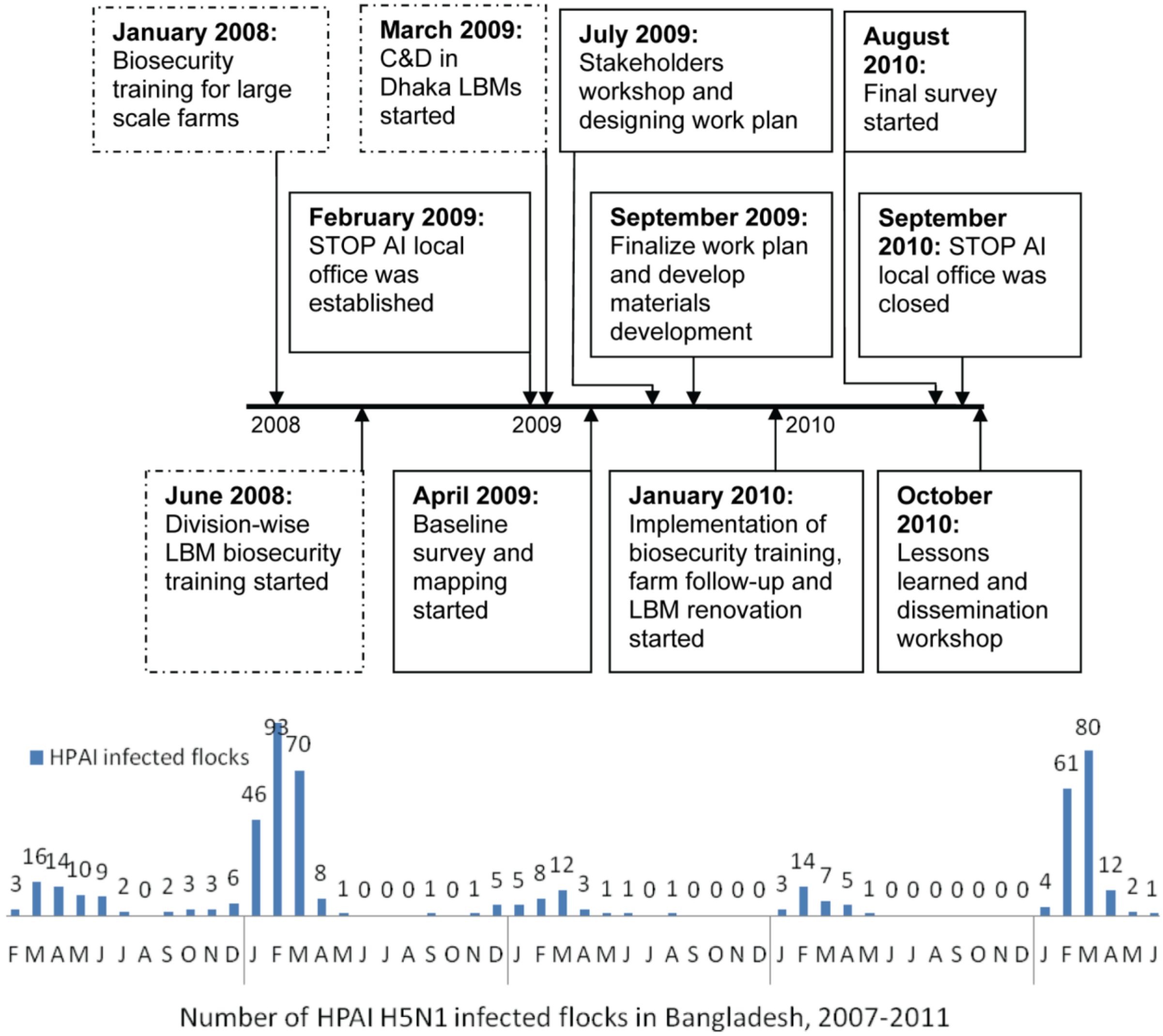

Figure 1. Timeline showing key events of Stamping Out Pandemic and Avian Influenza (STOP AI) project activities, 2008–2010, and weekly number of highly pathogenic avian influenza (HPAI) subtype H5N1 virus outbreaks, Bangladesh, 2007–2011. The activities within dashed-dotted areas are not included in this poultry value chain study. Significantly less numbers of outbreaks were reported during and after the STOP AI intervention. Revised HPAI control strategy (active surveillance and culling) implemented by the government of Bangladesh during that period also may have helped to reduce the number of HPAI outbreaks (*1*). C&D, cleaning and disinfection; LBM, live bird market.

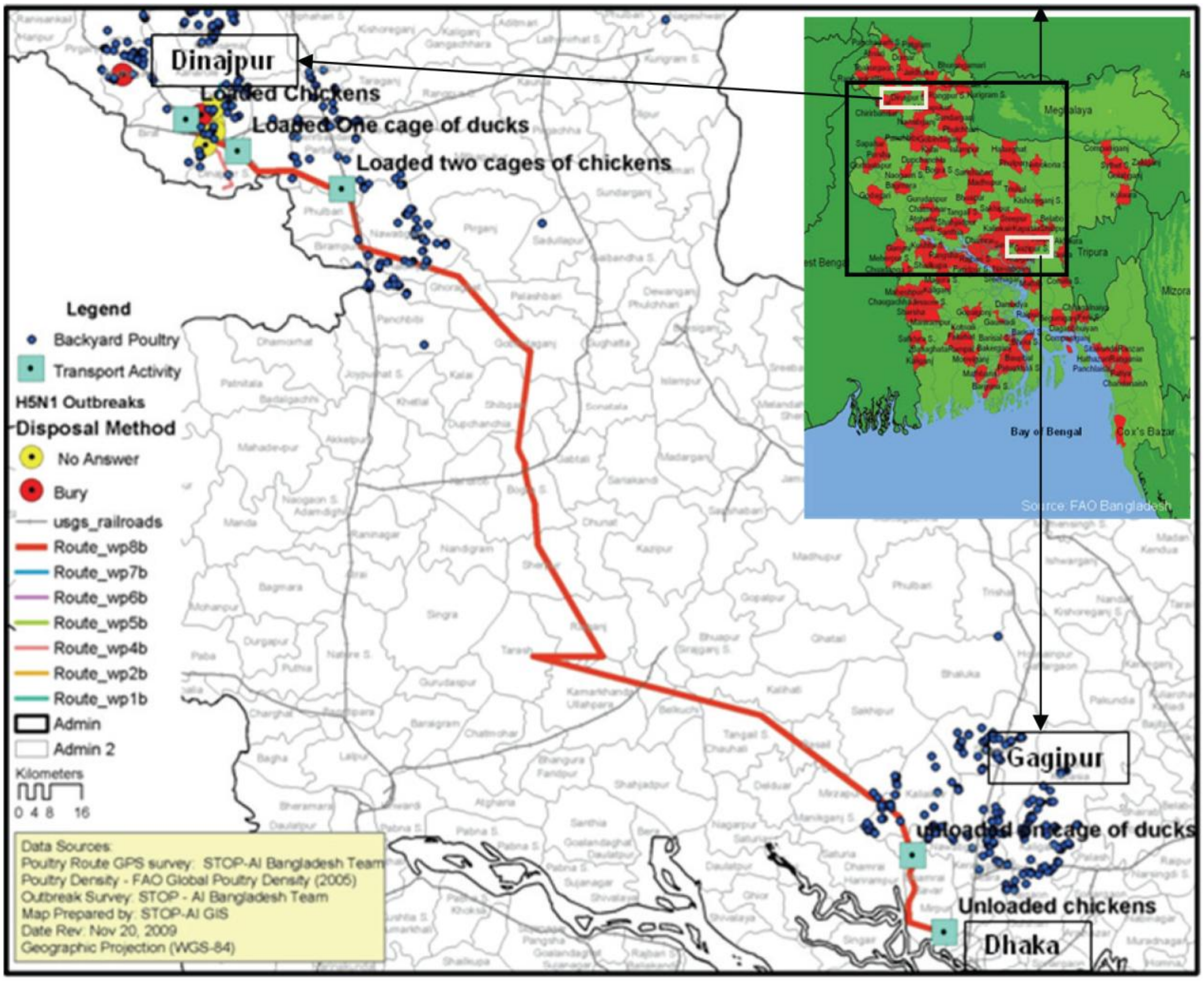

Figure 2. Map of Bangladesh showing locations of intervention districts, Gazipur and Dinajur, 2009–2010, and highly pathogenic avian influenza (HPAI) subtype H5N1 virus outbreaks (red clusters on right top), 2007–2011. Geographic positioning system coordinates were recorded by using personal navigators (eTrex Venture, Garmin, Olathe, KS, USA), and a geographic information system program (Arc View 9.1; Environmental System Research Institute, Redlands, CA, USA) was used to analyze data. Some outbreak farms (large circles) in Dinajpur and backyard poultry (small circles) in Gazipur and Dinajpur illustrate the functional relationship among poultry value chain stakeholders in two districts. Movement of poultry from Dinajpur to Dhaka (red line) by public buses and transport activities (squares on red line) could identify patterns of risk. Gazipur and Dinajpur are located in the northwestern and central parts of Bangladesh, respectively, and both are on an oblique line connecting with southeastern part of the country. The highway connecting this line is the main poultry trading route in the country (*2*). Epidemiologic studies have shown that the highest intensity and magnitude of the subtype H5N1 outbreaks was systematic and persistent in this line (*1,3*). Previous studies have confirmed the role of the highway network in HPAI (H5N1) epidemiology in other countries (*4,5*).

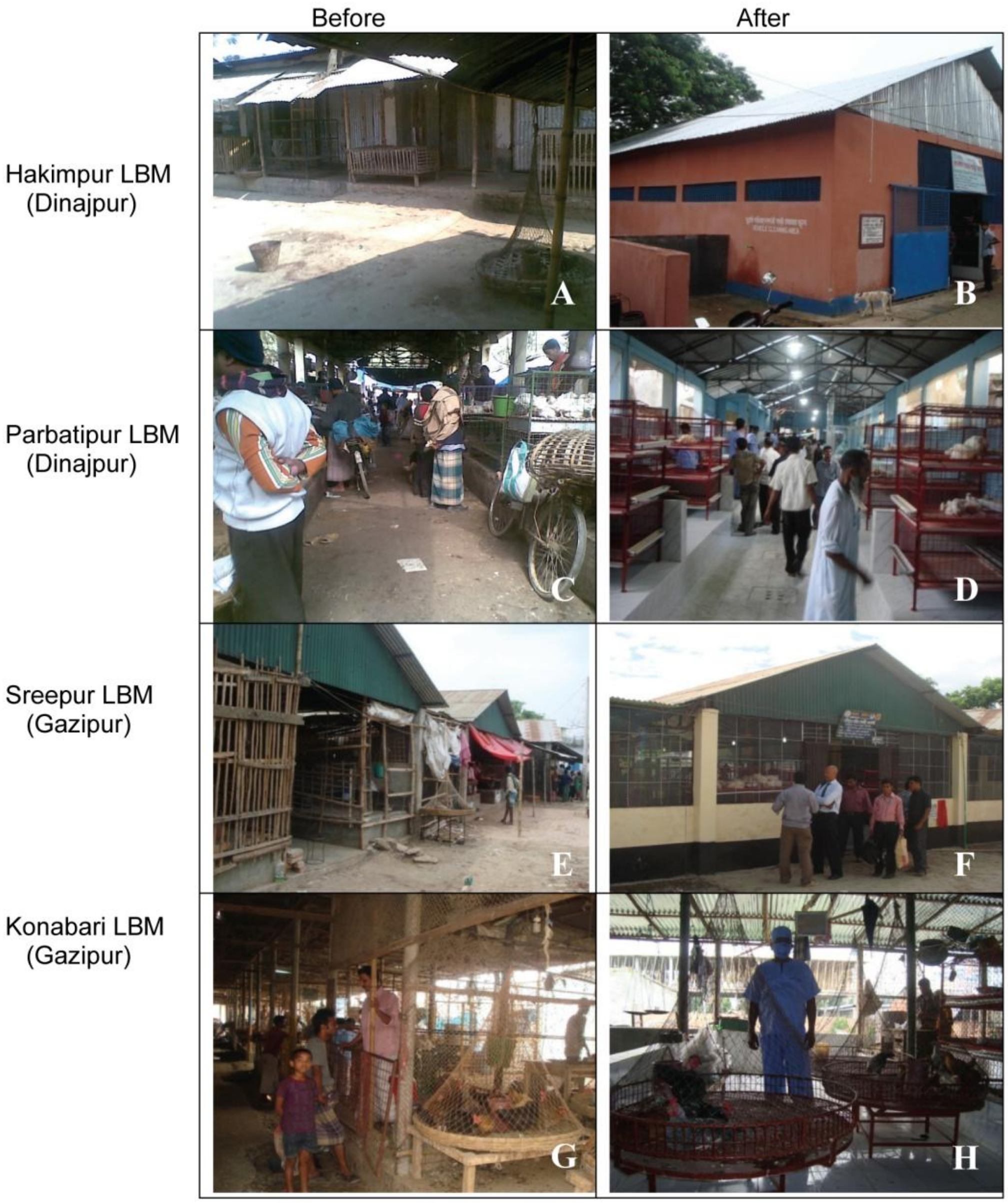

Figure 3. Infrastructure improvements in live bird markets (LBMs) in Dinajpur and Gazipur districts, Bangladesh, 2010. After assessing several LBMs in each district, 2 were selected in Dinajpur (A, C) and 2 in Gazipur (E, G) for our intervention. The renovated Hakimpur (B) and Parbatipur (D) LBMs in Dinajpur, and Sreepur (F) and Konabari LBMs (H) in Gazipur included tiling of poultry stalls and floors; supplying metal cages, pressure sprayer for cleaning and disinfection, improved water supply and drainage, modern slaughter facilities for hygienic processing of poultry meat; and establishing compost and biogas for waste disposal. In all 4 markets, biogas plants are being used for proper disposal of LBM waste and large animal slaughter waste, and the connected generators are being used to run lights and fans on a regular basis because frequent electric failure (load shedding) is common in Bangladesh.

References

1. Loth L, Gilbert M, Osmani MG, Kalam AM, Xiao X. Risk factors and clusters of highly pathogenic avian influenza H5N1 outbreaks in Bangladesh. Prev Vet Med. 2010;96:104–13.
2. Dolberg, F. Poultry sector country review: Bangladesh. Rome: Food and Agriculture Organization of the United Nations; 2009.
3. Ahmed SS, Ersbøll AK, Biswas PK, Christensen JP, Toft N. Spatio-temporal magnitude and direction of highly pathogenic avian influenza (H5N1) outbreaks in Bangladesh. PLoS One. 2011;6:e24324.
4. Rivas AL, Chowell G, Schwager SJ, Fasina FO, Hoogesteijn AL, Smith SD, et al. Lessons from Nigeria: the role of roads in the geo-temporal progression of avian influenza (H5N1) virus. Epidemiol Infect. 2010;138:192–8.
5. Ward MP, Maftei D, Apostu C, Suru A. Geostatistical visualisation and spatial statistics for evaluation of the dispersion of epidemic highly pathogenic avian influenza subtype H5N1. Vet Res. 2008;39:22.
